# Preoperative serum CA125 level is a good prognostic predictor in patients with intrahepatic cholangiocarcinoma after hepatectomy: A single-center retrospective study

**DOI:** 10.1097/MD.0000000000034839

**Published:** 2023-09-08

**Authors:** Jie Meng, Jun Weng, Jian Wu, Han Mao, Peilu Huang, Shule Chen, Lingyun Liu

**Affiliations:** a Department of Health Management Center, The Affiliated Hospital of Guilin Medical University, Guilin, China; b Department of Hepatobiliary and Pancreatic Surgery, The Affiliated Hospital of Guilin Medical University, Guilin, China; c Department of Hepatic Surgery, The First Affiliated Hospital of Sun Yat-Sen University, Guangzhou, China; d Department of Hepatobiliary and Pancreatic Surgery, Laboratory of Hepatobiliary and Pancreatic Surgery, Guangxi Key Laboratory of Molecular Medicine in Liver Injury and Repair, The Affiliated Hospital of Guilin Medical University, Guilin, China.

**Keywords:** biomarkers, CA125 antigen, hepatectomy, intrahepatic cholangiocarcinoma, prognosis

## Abstract

Serum carbohydrate antigen 125 (CA125) is associated with the prognosis of various malignancies, including ovarian and pancreatic cancer. The relationship between preoperative serum CA125 level and the survival of patients with intrahepatic cholangiocarcinoma (ICC) has not been fully studied. The aim of this study was to explore the prognostic value of CA125 in ICC after hepatectomy. We retrospectively reviewed the clinicopathological data of 178 ICC patients who underwent hepatic resection. Receiver operating characteristic analyses were performed to estimate the relationships of serum CA125, α-fetoprotein, carcinoembryonic antigen (CEA), and carbohydrate antigen 19-9 with the prognosis of ICC. The predictive value of CA125 for the prognosis of ICC patients was demonstrated by univariate analyses and Cox proportional hazards models. CA125 was correlated with tumor size, differentiation, capsulation, tumor node-metastasis stage, recurrence, and CEA. Univariate analysis indicated that CA125, sex, tumor number, tumor size, differentiation, surgical resection margin, tumor node metastasis stage, and CEA were risk factors for both the overall survival and the disease-free survival of ICC patients. Cox proportional hazards models showed that preoperative elevated CA125, a tumor size > 5 cm, and an R1 surgical resection margin were independent prognostic predictors of overall survival and disease-free survival. CA125 also had strong predictive value for the prognosis of different ICC subgroups, including patients without lymph node metastasis and with elevated carbohydrate antigen 19-9 levels. Preoperative elevated serum CA125 level is a noninvasive, simple, and reliable indicator of the prognosis of ICC patients after hepatectomy.

## 1. Introduction

Intrahepatic cholangiocarcinoma (ICC) is the second most common hepatic malignancy after hepatocellular carcinoma.^[1]^ The incidence of ICC has progressively increased in recent decades.^[2,3]^ Most patients with ICC are diagnosed at an advanced stage because of the lack of specific clinical manifestations.^[4,5]^ Complete surgical resection is the only way to achieve curative therapeutic outcomes for patients with ICC. Unfortunately, the 5-year survival rate of ICC patients who undergo curative hepatectomy ranges from 25% to 40%, and early tumor recurrence and metastasis are the main reasons for the grim clinical outcomes of ICC patients after surgery.^[6]^ Therefore, there is an urgent need to screen for effective tumor biomarkers that would identify ICC patients with a high risk of tumor recurrence or metastases, which could reveal further treatment strategies to improve their prognosis.

Carbohydrate antigen 125 (CA125) is a classical tumor marker for the diagnosis and monitoring of ovarian tumors.^[7]^ Recently, several studies have indicated that serum CA125 level is related to the prognosis of various malignancies, including ovarian cancer^[8]^ and cervical adenocarcinoma.^[9]^ In 2012, Michiyo et al^[10]^ evaluated the expression of MUC16 (a glycoprotein that carries the peptide epitope CA125) in 63 ICC–mass-forming type tissues, and the results showed that MUC16/CA125 expression in tumor tissues was a prognostic predictor for poor survival of ICC–mass-forming type.

To our knowledge, the relationship between serum CA125 level and the prognosis of patients with ICC after hepatectomy have not been explored yet. In this study, we aimed to explore the prognostic significance of pretreatment serum CA125 level on survival rates in ICC patients.

## 2. Materials and methods

### 2.1. Study population

A total of 178 ICC patients treated with hepatic resection were admitted to the First Affiliated Hospital of Sun Yat-sen University from April 2004 to September 2015. All specimens were histologically diagnosed as ICC after surgery based on the 2010 classification of tumors of the digestive system (World Health Organization).^[11]^ Written informed consent was waived because of the retrospective nature of this study, and this study was given prior approval by the ethics committee of our hospital. Prior to surgery, a routine assessment was carried out for all patients, including a complete physical examination, hematologic and biochemistry tests, electrocardiograph, chest X-ray, abdominal ultrasound (including ultrasound contrast), and computed tomography (CT) or magnetic resonance imaging scans.

All patients were more than 18 years old and had complete clinical, pathological and laboratory data. There were no coexisting disorders or specific physiological stages (such as menstrual periods or pregnancy in female patients) that impacted serum CA125 level before treatment. No patient had received antitumor treatment before surgery, such as chemoradiotherapy or percutaneous ablation.

### 2.2. Treatment and follow-up

Operation strategies were designed in routine multidisciplinary team meetings. Combined operations that involved the bile duct, pancreas and stomach were applied when needed. All 178 patients received regular postoperative follow-up according to institutional practice, including liver ultrasound, chest X-ray and assessment of serum CA125, carbohydrate antigen 19-9 (CA19-9), and carcinoembryonic antigen (CEA) every 3 months, plus contrast-enhanced CT scans every 6 months. Tumor recurrence was diagnosed by clinical manifestation and by radiological and/or pathological tests (on mass tissues obtained by ultrasound- or CT-guided fine-needle aspiration). Patients with confirmed tumor relapse received salvage treatments, such as chemoradiotherapy, repeated hepatectomy, and radiofrequency ablation. The disease-free survival (DFS) time was calculated from the date of surgery to the date of tumor recurrence, and the overall survival (OS) time was calculated from the date of surgery to the date of ICC-associated death or last follow-up date. The last date of follow-up was August 3, 2018.

### 2.3. Statistical analysis

The statistical analyses were conducted with SPSS version 22.0 software (Chicago, IL). Receiver operating characteristic (ROC) curves were drawn to assess the difference in the area under the ROC curve. The Mann–Whitney *U* test was performed to evaluate nonnormally distributed numerical variables. The Pearson chi-square test was used to compare the differences in categorical variables, and correlations between these variables were tested by Pearson bivariate correlate analyses. Survival analyses (including stratified analysis) were performed with the Kaplan–Meier method and were compared by the log-rank test. The Cox proportional hazards model (backward stepwise) was used to screen for independent prognostic indicators. *P* < .05 (2-tailed tests) was considered significant.

## 3. Results

### 3.1. Patient characteristics

The study included 91 males (51.1%) and 87 females (48.9%) with a median age of 56 years (ranging from 24 to 82 years). A total of 159 patients (89.3%) developed tumor relapse, and 148 patients (83.1%) died during the follow-up period. Cirrhosis existed in 43 patients (24.2%), and 47 patients (26.4%) were serum hepatitis B surface antigen (HBsAg)-positive. Twenty-four patients had a past history of biliary tract surgery on account of hepatolithiasis. The median longest tumor diameter was 6.0 cm (ranging from 1.0–20.0 cm), and multiple tumor masses existed in 63 patients (35.4%). Based on the World Health Organization 2010 classifications regarding tumor differentiation,^[11]^ 120 patients (67.4%) had a well/moderately differentiated status, and 58 patients (32.6%) had a poorly differentiation status. According to the AJCC staging manual (American Joint Committee on Cancer) 8^th^ edition,^[12]^ 72 patients (40.4%) were in tumor node metastasis (TNM) stage I to II, and 106 patients (59.6%) were in TNM stage III to IV. Increased CA19-9 levels (> 35 U/mL) were found in 120 patients (67.4%). The cutoff level of serum CA125 was 35 U/mL (according to routine clinical testing in our hospital). In addition, all 178 cases were divided into a high-CA125 group (> 35 U/mL, n = 80) and a low-CA125 group (≤ 35 U/mL, n = 98). The clinical and pathological characteristics of all patients are listed in Table 1.

**Table 1:** Correlation between preoperative serum CA125 and clinicopathological characteristics of ICC (n = 178).

Category	Subcategory	No	CA125 (U/mL)		*χ^2^*	*r*	*P* value
			≤ 35.0 (n = *98*)	> 35.0 (n = *80*)			
Age (yr)	≤ 60	115	65 (56.5%)	50 (43.5%)	0.282		.595
	> 60	63	33 (52.4%)	30 (47.6%)			
Sex	Female	87	51 (58.6%)	36 (41.4%)	0.874		.350
	Male	91	47 (51.6%)	44 (48.4%)			
Preoperative symptom	No	49	32 (65.3%)	17 (34.7%)	2.871		.090
	Yes	129	66 (51.2%)	63 (48.8%)			
Cirrhosis	No	135	71 (52.6%)	64 (47.4%)	1.371		.242
	Yes	43	27 (62.8%)	16 (37.2%)			
HBsAg	Negative	131	71 (54.2%)	60 (45.8%)	0.148		.701
	Positive	47	27 (57.4%)	20 (42.6%)			
Child-Pugh Class	A	156	90 (57.7%)	66 (42.3%)	3.545		.060
	B	22	8 (36.4%)	14 (63.6%)			
Tumor number	Single	115	69 (60.0%)	46 (40.0%)	3.209		.073
	Multiple	63	29 (46.0%)	34 (54.0%)			
Tumor size (cm)	≤ 5	65	48 (73.8%)	17 (26.2%)	14.609	0.286	<.001
	> 5	113	50 (44.2%)	63 (55.8%)			
Differentiation*	Well/moderate	120	74 (61.7%)	46 (38.3%)	6.504	0.191	.011
	poor	58	24 (41.4%)	34 (58.6%)			
Capsulation	No	108	53 (49.1%)	55 (50.9%)	3.972	−0.149	.046
	Yes	70	45 (64.3%)	25 (35.7%)			
Resection margin	R0	101	60 (59.4%)	41 (40.6%)	1.785		.182
	R1	77	38 (49.4%)	39 (50.6%)			
TNM†	I-II	72	52 (72.2%)	20 (27.8%)	14.398	0.284	<.001
	III-IV	106	46 (43.4%)	60 (56.6%)			
Lymph node metastasis	No	106	66 (62.3%)	40 (37.7%)	5.502	0.176	.019
	Yes	72	32 (44.4%)	40 (55.6%)			
Distant metastasis	No	141	85 (60.3%)	56 (39.7%)	7.491	0.205	.006
	Yes	37	13 (35.1%)	24 (64.9%)			
Recurrence	No	19	17 (89.5%)	2 (10.5%)	10.183	0.239	.001
	Yes	159	81 (50.9%)	78 (49.1%)			
AFP (μg/L)	≤ 200	171	94 (55.0%)	77 (45.0%)	0.013		.910
	> 200	7	4 (57.1%)	3 (42.9%)			
CEA (μg/L)	≤ 5	99	64 (64.6%)	35 (35.4%)	8.291	0.216	.004
	> 5	79	34 (43.0%)	45 (57.0%)			
CA19-9 (U/mL)	≤ 35	58	36 (62.1%)	22 (37.9%)	1.710		.191
	> 35	120	62 (51.7%)	58 (48.3%)			

AFP = alpha fetal protein, CA125 = carbohydrate antigen 125, CA19-9 = carbohydrate antigen 19-9, CEA = carcinoembryonic antigen, HBsAg = hepatitis B surface antigen, ICC = intrahepatic cholangiocarcinoma, TNM = tumor node metastasis.

* According to the World Health Organization (WHO) classification of tumors of the digestive system 2010.

† Based on the eighth edition of the cancer staging manual of the American Joint Committee on Cancer.

### 3.2. Association of preoperative CA125 with clinicopathological parameters in ICC

The results indicated that preoperative serum CA125 was associated with tumor size (χ^2^ = 14.609, *P* < .001), differentiation (χ^2^ = 6.504, *P* = .011), tumor capsulation (χ^2^ = 3.972, *P* = .046), TNM stage (χ^2^ = 14.398, *P* < .001), lymph node metastasis (χ^2^ = 5.502, *P* = .019), distant metastasis (χ^2^ = 7.491, *P* = .006), tumor recurrence (χ^2^ = 10.183, *P* = .001) and CEA (χ^2^ = 8.291, *P* = .004). There were no associations of CA125 with age, sex, preoperative symptoms, cirrhosis, HBsAg positivity, tumor number, resection margin, alpha fetal protein (AFP) or CA19-9 (all *P* > .05, Table 1).

### 3.3. Serum CA125 levels predict clinical outcomes more precisely than AFP, CEA, or CA19-9

**Figure 1.**
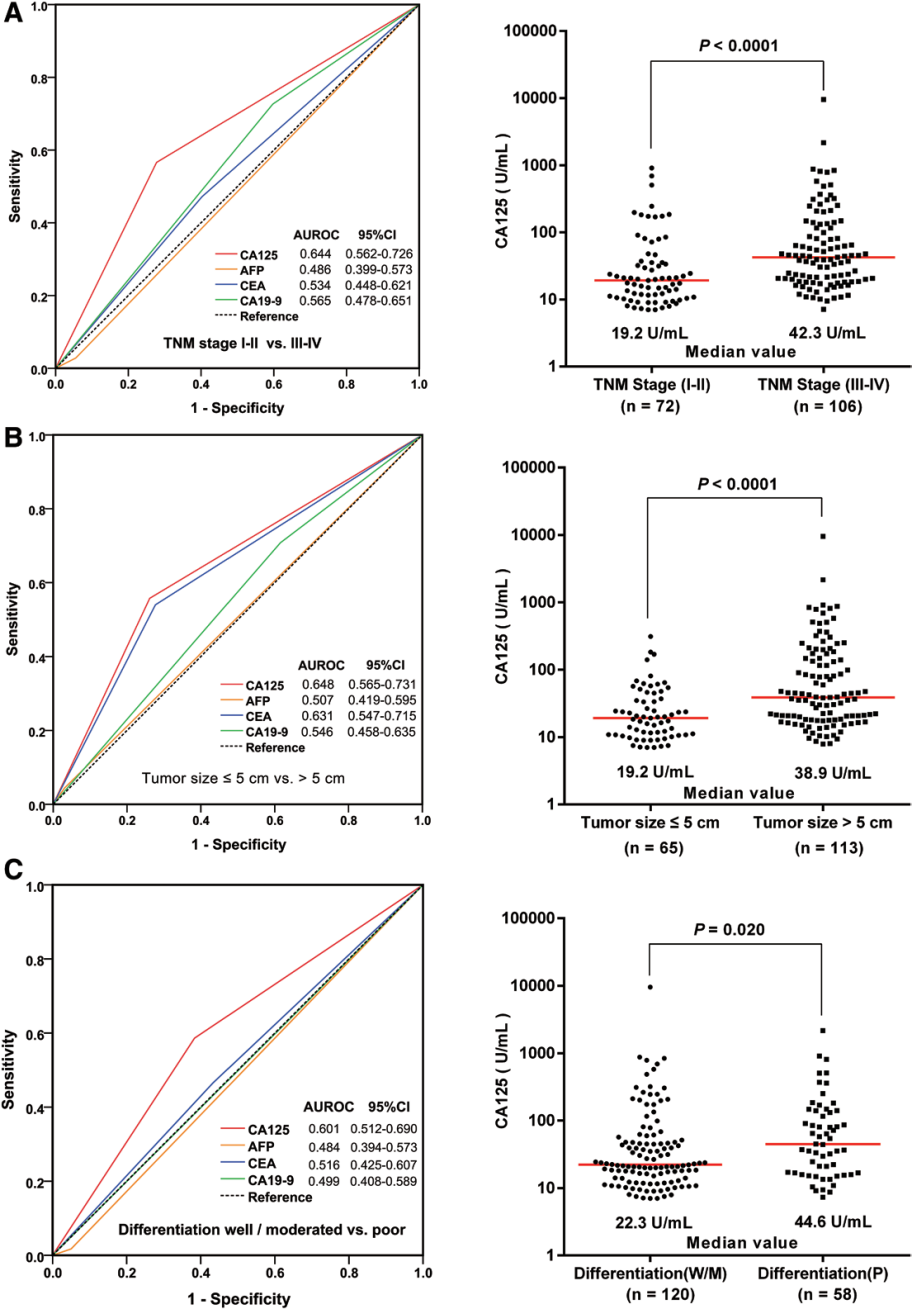
Receiver operating characteristic (ROC) curve analysis showed that carbohydrate antigen 125 (CA125) was more accurate than α-fetoprotein (AFP), carcinoembryonic antigen (CEA), or carbohydrate antigen 19-9 (CA19-9) in predicting the prognosis of various intrahepatic cholangiocarcinoma (ICC) subgroups and the distribution of CA125 levels in the different subgroups. (A) For TNM stage (I–II vs III–IV), the area under the ROC curve (AUROC) of CA125 was 0.644 (95% CI 0.562–0.726, *P* < .001). (B) For tumor size (cm, ≤ 5 vs > 5), the AUROC of CA125 was 0.648 (95% CI 0.565–0.731, *P* < .001). (C) For tumor differentiation (well/moderate vs poor), the AUROC of CA125 was 0.601 (95% CI 0.512–0.690, *P* = .020). The proportions of ICC patients with elevated CA125 along with TNM stage III–IV, tumor size > 5 cm, and poor differentiation were higher than those of their counterpart subgroups (all *P* < .05, Mann–Whitney U test). Serum CA125 level (log10 scale) was plotted on the y-axis. The lines across the dot plots represent median values. AUROC = the area under the ROC curve, CI = confidence interval, TNM = tumor node metastasis.

Based on TNM stage, tumor size classification and differentiation, ROC curve analyses indicated that CA125 was a more sensitive serological tumor indicator of clinical outcomes than AFP, CEA, or CA19-9 (all *P* < .05, Fig. 1A–C). Patients with TNM stage I-II disease had lower serum CA125 levels than those with TNM stage III-IV disease (*P* < .001) (Fig. 1A). A similar tendency was also present in ICC patients with a tumor size ≤ 5 cm and well/moderate tumor differentiation compared to those with a tumor size > 5 cm and poor differentiation (*P* < .001 and *P* = .020, respectively) (Fig. 1B and C).

### 3.4. Risk factors for OS and DFS of ICC patients

The 1-year and 3-year OS rates for all ICC patients were 38.2% and 13.4%, respectively. In addition, the 1-year and 3-year DFS rates for the entire cohort were 22.5% and 10.7%, respectively. Univariate analysis identified preoperative CA125, sex, tumor number, tumor size, tumor differentiation, resection margin, TNM stage, and CEA as prognostic indicators for both OS and DFS of ICC patients (all *P* < .05, Table 2). Next, multivariate Cox proportional hazard regression analysis indicated that CA125, tumor size and resection margin were independent prognostic indicators of OS and DFS (all *P* < .05), while tumor differentiation was an independent predictor for OS only (*P* < .05) (Table 3).

**Table 2:** Prognostic risk factors for OS and DFS of ICC patients by univariate analysis (n = 178).

Category	No	OS		*P* value	DFS		*P* value
		1-yr	3-yr		1-yr	3-yr	
Age (yr)				.676			.479
≤ 60	115	34.8%	14.2%		20.9%	10.3	
> 60	63	44.4%	9.4%		25.4%	11.8%	
Sex				.010			.029
Female	87	47.1%	21.2%		29.9%	14.1%	
Male	91	29.7%	6.2%		15.4%	7.7%	
Preoperative symptom							
Absent	49	46.9%	20.4%	.177	32.7%	9.8%	.162
Present	129	34.9%	11.0%		18.6%	10.6%	
Cirrhosis				.595			.353
Negative	135	40.0%	11.4%		23.7%	9.3%	
Positive	43	32.6%	12.4%		18.6%	13.3%	
HBsAg				.780			.386
Negative	131	38.9%	14.1%		24.4%	11.3%	
Positive	47	36.2%	11.6%		17.0%	5.7%	
Child-pugh class				.526			.311
A	156	37.2%	12.7%		21.2%	9.6%	
B	22	45.5%	18.2%		31.8%	18.2%	
Tumor number				.024			.013
Single	115	43.5%	14.8%		27.0%	11.6%	
Multiple	63	28.6%	11.0%		14.3%	8.9%	
Tumor size (cm)				<.001			<.001
≤ 5	65	53.8%	25.8%		41.5%	20.3%	
> 5	113	29.2%	6.5%		11.5%	4.9%	
Tumor differentiation				.001			.013
Well/moderate	120	44.2%	17.6%		26.7%	12.7%	
Poor	58	25.9%	4.0%		13.8%	7.1%	
Capsulation				.184			.564
No	108	36.1%	8.7%		22.2%	7.2%	
Yes	70	41.4%	18.4%		22.9%	14.9%	
Resection margin				.003			.004
R0	101	43.6%	20.5%		27.7%	17.5%	
R1	77	31.2%	3.8%		15.6%	4.3%	
TNM				.006			.010
I–II	72	47.2%	21.4%		31.9%	20.4%	
III–IV	106	32.1%	7.7%		16.0%	4.2%	
AFP (μg/L)				.893			.417
≤ 200	171	38.0%	13.4%		22.8%	10.4%	
> 200	7	42.9%	14.3%		14.3%	0.0%	
CEA (μg/L)				.003			.001
≤ 5	99	46.5%	20.0%		30.3%	15.5%	
> 5	79	27.8%	4.6%		12.7%	4.3%	
CA19-9 (U/mL)				.160			.265
≤ 35	58	46.6%	13.6%		25.9%	9.4%	
> 35	120	34.2%	13.4%		20.8%	11.1%	
CA125 (U/mL)							
≤ 35	98	50.0%	20.1%	<.001	31.6%	17.9%	<.001
> 35	80	23.8%	4.9%		11.2%	2.1%	

AFP = alpha fetal protein, CA19-9 = carbohydrate antigen 19-9, CA125 = carbohydrate antigen 125, CEA = carcinoembryonic antigen, DFS = disease-free survival, HBsAg = hepatitis B surface antigen, ICC = intrahepatic cholangiocarcinoma, OS = overall survival, TNM = tumor node metastasis.

**Table 3:** Independent prognostic factors of ICC patients by multivariate Cox analysis (n = 178).

Variables	OS		DFS	
	HR (95% CI)	*P* value	HR (95% CI)	*P* value
Tumor differentiation (well/moderate vs poor)	1.629 (1.156–2.297)	.005		
Tumor size (cm, ≤ 5 vs > 5)	1.676 (1.168–2.407)	.005	1.914 (1.341–2.732)	<.001
Resection margin (R0 vs R1)	1.558 (1.122–2.165)	.008	1.496 (1.087–2.059)	.013
CA125 (U/mL, ≤ 35 vs > 35)	1.841 (1.309–2.589)	<.001	1.547 (1.110–2.156)	.010

Variables with *P* < .05 calculated by Kaplan–Meier methods (log-rank test) were used in a multivariate Cox proportional hazards regression model (backward stepwise).

CA125 = carbohydrate antigen 125, CI = confidence interval, DFS = disease-free survival, HR = hazard ratio, ICC = intrahepatic cholangiocarcinoma, OS = overall survival.

### 3.5. Preoperative serum CA125 levels predicted OS and DFS

**Figure 2.**
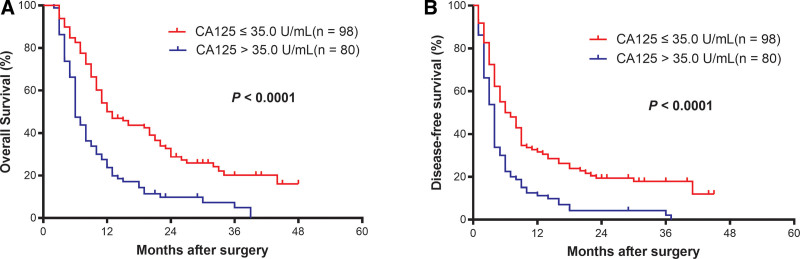
Survival curves of intrahepatic cholangiocarcinoma patients with elevated and normal serum CA125 level in the entire cohort (all *P* < .0001). *P* values were obtained by Kaplan–Meier methods and log-rank tests. CA125 = carbohydrate antigen 125.

Kaplan–Meier analysis indicated that the 1-year and 3-year OS rates of the CA125 ≤ 35 group were significantly higher than those of the CA125 > 35 group (50.0% and 20.1% vs 23.8% and 4.9%, respectively, *P* < .001) (Table 2, Fig. 2A). Similar phenomena were found in the 1-year and 3-year DFS rates of ICC patients with normal or elevated CA125 (31.6% and 17.9% vs 11.2% and 2.1%, respectively, *P* < .001) (Table 2, Fig. 2B). These observations show that preoperative serum CA125 level was a good survival indicator in ICC patients after hepatectomy.

### 3.6. Stratified analysis in subgroups of ICC according to CA125

**Figure 3.**
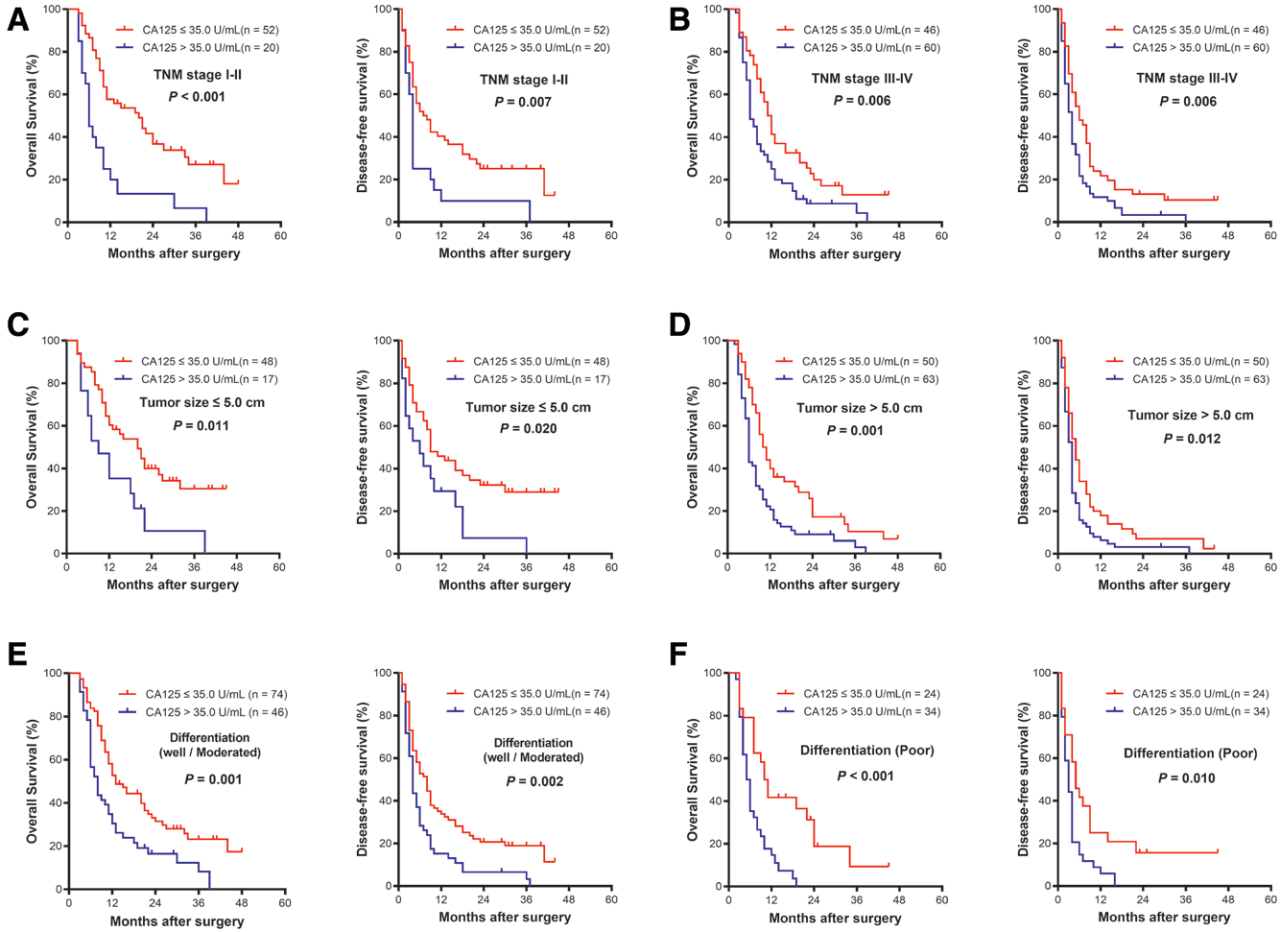
Kaplan–Meier survival curves of patients with intrahepatic cholangiocarcinoma after hepatic resection stratified by TNM stage I–II (A), TNM stage III–IV (B), tumor size (cm, ≤ 5) (C), tumor size (cm, > 5) (D), differentiation (well/moderate) (E), and poor differentiation (F) (all *P* < .05). TNM = tumor node metastasis.

**Figure 4.**
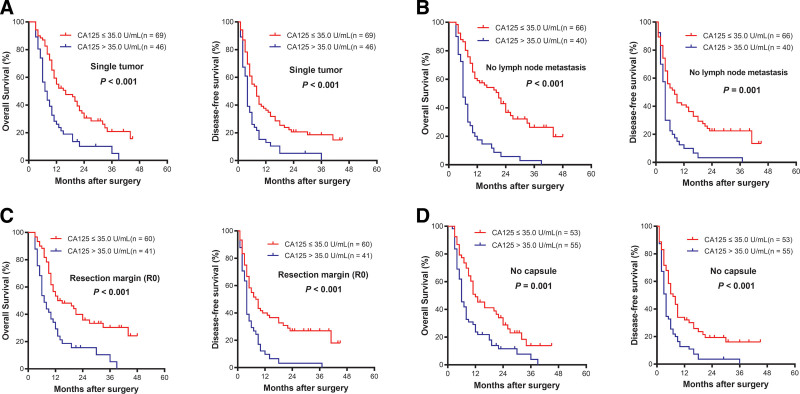
Survival curves of patients with intrahepatic cholangiocarcinoma stratified by single-tumor status (A), no lymph node metastasis (B), R0 resection margin **(C**), and no capsule (D) (all *P* < .05).

**Figure 5.**
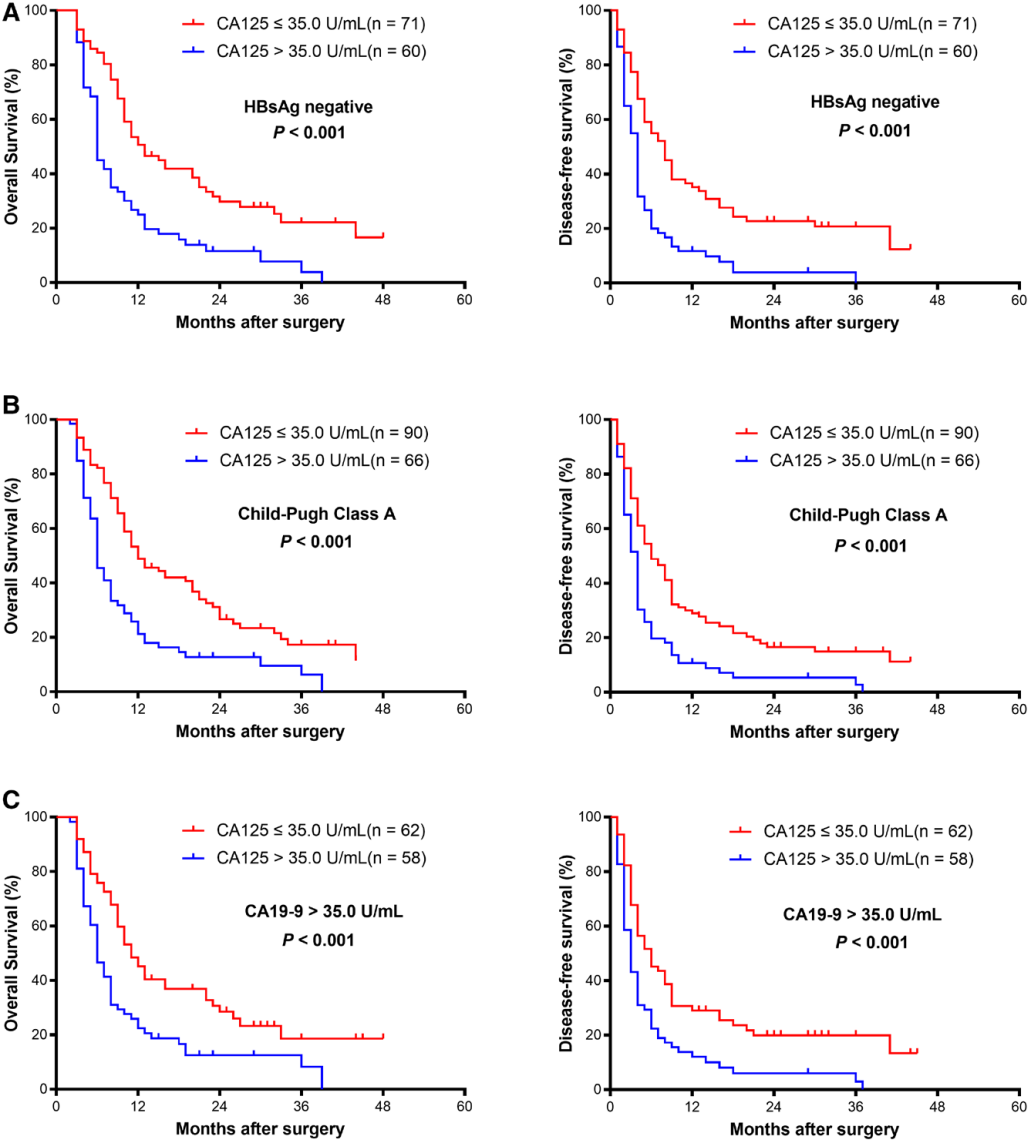
Kaplan–Meier survival curves of patients with intrahepatic cholangiocarcinoma stratified by serum HBsAg negativity (A), Child–Pugh Class A (B), and CA19-9 > 35 U/mL (C) (all *P* < .001). CA19-9 = carbohydrate antigen 19-9.

The results above confirmed that serum CA125 was associated with the prognosis of the entire ICC cohort. Further subgroup analyses suggested that preoperative serum CA125 level served as a good prognostic indicator for both OS (*P* < .001) and DFS (*P* = .007) in ICC patients with TNM stage I-II disease (Fig. 3A). Similarly, preoperative serum CA125 showed remarkable predictive value of the prognosis in the subgroup of ICC patients with TNM stage III to IV, tumor size ≤ 5 cm, tumor size > 5 cm, well/moderate differentiation, poor differentiation, single-tumor, no lymph node metastasis, R0 surgical resection margin, no tumor capsule, HBsAg negative status, Child–Pugh class A and CA19-9 > 35.0 U/mL (all *P* < .05) (Figs. 3B–F, 4A–D, and 5A–C). These results demonstrated that CA125 was a sensitive prognostic indicator for ICC patients who have undergone hepatectomy, including subgroups of ICC patients whose prognoses were difficult to predict.

## 4. Discussion

Currently, ICC patients have a poor prognosis even if they undergo radical resection. Finding a simple and reliable biomarker to identify ICC patients at high risk of tumor recurrence and metastasis after hepatectomy is of great importance to improve their clinical outcomes. In the present study, we found that preoperative serum CA125 is a noninvasive and effective prognostic indicator in patients with ICC after hepatectomy.

Previous studies have confirmed that serum CA125 is a predictor of prognosis in various malignancies, including epithelial ovarian cancer,^[7]^ cervical adenocarcinoma,^[9]^ and endometrial carcinoma.^[13]^ CA125 is a transmembrane mucin encoded by the mucin 16 (MUC16) gene.^[14]^ CA125/MUC16 might also play an important role in the tumorigenesis and metastasis of pancreatic cancer^[15]^ and ovarian tumors.^[16]^ Hence, we assessed the relationship between pretreatment CA125 and the clinical outcomes of ICC patients after hepatic resection. We found that preoperative serum CA125 is a good prognostic indicator for ICC patients. Patients with elevated CA125 (> 35 U/mL) had earlier tumor relapse and shorter overall survival times.

Our findings suggested that CA125 was positively correlated with tumor size, differentiation, tumor capsulation, and TNM stage, which means that ICC patients with elevated CA125 might have a larger tumor burden and earlier recurrence or metastasis. Survival analysis indicated that tumor size > 5 cm, R1 surgical resection margin and elevated CA125 were poor independent predictors of both OS and DFS in patients with ICC after hepatectomy, while poor differentiation was only an independent risk factor for OS.

More in-depth stratification analysis suggested that CA125 was also a good prognostic indicator in TNM stage I to II ICC patients; that is, preoperative serum CA125 level could predict the tumor relapse of early-stage ICC. A histologically positive resected margin is a risk factor in the prognosis of ICC patients.^[17,18]^ It is generally believed that it is more difficult to assess the prognosis of ICC patients with R0 resected margins after surgery. However, the results of this study showed that CA125 had a significant ability to predict the prognosis of ICC patients. Furthermore, in subgroups of ICC patients with TNM stage III to IV, tumor size ≤ 5 cm, tumor size > 5 cm, well/moderate differentiation, poor differentiation, single-tumor, no lymph node metastasis, no capsule, serum HBsAg negativity, Child–Pugh Class A and an elevated serum CA19-9 level, preoperative elevated CA125 also showed value as a predictor of poorer OS and DFS. The results indicate that the preoperative serum CA125 level is a potentially reliable prognostic predictor in ICC patients after hepatic resection.

The data of this study showed that elevated CA125 was correlated with advanced TNM stage and aggressive tumor biological behaviors and that preoperative serum CA125 level is a good prognostic indicator for ICC patients. The exact reasons for this phenomenon remain largely unclear. As a very large transmembrane mucin, CA125/MUC16 is widely used to monitor patients with ovarian cancer.^[14,19]^ Several studies have also reported that CA125/MUC16 is overexpressed in various malignancies, including pancreatic ductal adenocarcinoma and gastric and colon cancer.^[20,21]^ Furthermore, CA125/MUC16 is considered one of the 3 most frequently mutated genes in many types of tumors.^[22]^ CA125/MUC16 showed oncogenic potential in transforming ovarian cells.^[16]^ Another study verified that the carboxyl-terminal polypeptide fragment of MUC16 combined with stathmin1 promotes the migration and invasion of gallbladder cancer.^[23]^ It follows that CA125/MUC16 could play an important role in the tumorigenesis and metastasis of ICC. Treatment trials that targeted CA125/MUC16 have also shown promise. A human bispecific antibody (REGN4018)^[24]^ and Meso-TR3 chimera^[25]^ have the potential to kill MUC16-positive cancer cells. Therefore, based on our findings, CA125/MUC16 could be considered a potential therapeutic target for ICC patients with preoperative elevated CA125 levels.

The ROC analyses in this study indicated that serum CA125 level predicted clinical outcomes more accurately than routine serum AFP, CEA, and CA19-9 levels. Biomarkers of ICC, such as serum CA19-9 and CEA, have significant overlap in their concentrations with other, benign diseases, so their sensitivity and accuracy are far from satisfactory. For example, CA19-9 levels may be affected by acute cholangitis or bile duct obstruction. In this study, CA19-9 had no demonstrated prognostic value for the OS or DFS of ICC after hepatic resection. A study found that the serum cytokeratin-19 fragment (CYFRA 21-1) had higher specificities than CA19-9 for ICC but was not routinely used.^[26]^ Several studies have reported that oncogene and noncoding RNA expression are associated with ICC.^[27–29]^ There has not been enough evidence to support the use of gene testing for the diagnosis of ICC. Compared to these tumor markers, preoperative serum CA125 not only is more suitable for the highly accurate diagnosis and prognosis of ICC but is also simpler and cheaper because it is derived from routine preoperative examination.

Several limitations of the present study should be mentioned. It is a retrospective study with a relatively small sample size from a single-center in China. We were unable to randomly divide the patients into a training cohort and a validation cohort to obtain more accurate statistical results. Larger prospective multicenter studies should be carried out to confirm our findings.

## 5. Conclusion

We conclude that preoperative serum CA125 level is a good independent predictor of the prognosis of patients with ICC after liver resection. ICC patients with preoperative elevated CA125 might gain a survival benefit from close follow-up after surgery and accurate treatment.

## Author contributions

**Conceptualization:** Lingyun Liu.

**Data curation:** Lingyun Liu, Jian Wu, Peilu Huang, Shule Chen.

**Funding acquisition:** Lingyun Liu.

**Formal analysis:** Jie Meng, Jun Weng, Shule Chen.

**Investigation:** Jie Meng, Jun Weng, Jian Wu, Han Mao, Peilu Huang, Shule Chen.

**Methodology:** Lingyun Liu, Jie Meng, Jun Weng, Han Mao, Peilu Huang.

**Project administration:** Jun Weng.

**Resources:** Jian Wu.

**Software:** Lingyun Liu, Han Mao, Peilu Huang, Shule Chen.

**Validation:** Lingyun Liu, Jie Meng, Jian Wu, Han Mao.

**Writing – original draft:** Lingyun Liu.

**Writing – review & editing:** Lingyun Liu, Jie Meng.
